# Additional hospitalization costs associated with delirium among older adults: evidence from the Medicare Current Beneficiary Survey

**DOI:** 10.3389/fpubh.2026.1750969

**Published:** 2026-01-28

**Authors:** Emmanuel E. Garcia Morales, Mfon E. Umoh, Kayti Luu, Esther S. Oh, Nicholas S. Reed

**Affiliations:** 1 Optimal Aging Institute. Department of Population Health, NYU Grossman School of Medicine, New York, NY, United States; 2 Department of Otolaryngology-Head and Neck Surgery, NYU Grossman School of Medicine, New York, NY, United States; 3 Department of Medicine, Division of Geriatric Medicine and Gerontology, The Johns Hopkins University School of Medicine, Baltimore, MD, United States; 4 Department of Obstetrics and Gynecology, University of Hawaii John A Burns School Medicine, Honolulu, HI, United States; 5 Department of Psychiatry and Behavioral Sciences, The Johns Hopkins University School of Medicine, Baltimore, MD, United States; 6 Department of Pathology, The Johns Hopkins University School of Medicine, Baltimore, MD, United States; 7 The Johns Hopkins University School of Nursing, Baltimore, MD, United States

**Keywords:** Alzheimer’s disease, delirium, dementia, health care costs, healthcare utilization

## Abstract

**Introduction:**

Delirium, an acute state of confusion and inattention, affects over 7 million hospitalized adults in the United States (US) annually. Health care costs associated with delirium in the US have been estimated from prospective observational cohorts of hospitalized older adults, but these estimates may be skewed based on the community examined. Therefore, there is a need for estimates from nationally representative data.

**Methods:**

Additional expenditures associated with experiencing delirium during hospitalization were estimated after analyzing all inpatient claims from the 2019–2021 Medicare Current Beneficiary Survey (MCBS) research claim files. Hospital expenditure was calculated as the total charges for all services included in the institutional claim and total Medicare payments. Delirium during hospitalization was ascertained based on ICD-9 and ICD-10 codes from hospital claims data. We estimated the excess cost associated with delirium using a mixed-effects linear model with a random intercept at the individual level adjusting for demographic and health characteristics of patients.

**Results:**

In a sample of 2,599 Medicare beneficiaries (mean age 78.4 years, 52.9% female, 81.8% White), we identified a total of 5,677 hospitalization claims. A total of 286 (11.0%) participants experienced at least one delirium episode. In fully adjusted models, delirium was associated with $8,110 (95% confidence interval, CI: $1,860, $14,360) additional hospital charges and $1,631(95% CI: $527, $2,735) additional Medicare payments, compared to hospitalization without delirium. Delirium was more costly among participants with ADRD than those with no history of ADRD.

**Discussion:**

Findings from this nationally representative dataset show that delirium during hospitalization is associated with higher health care costs. As delirium is considered a modifiable risk factor for dementia, another condition with significant associated healthcare costs, these expenses may be preventable. Additional research is needed to understand the economic impact of delirium prevention and intervention.

## 1 Introduction

Delirium is a condition characterized by an acute disorder of cognition and attention; it is highly prevalent in hospitalized older adults (1–3). Delirium, though acute, can have long lasting implications and has been associated with other significant outcomes including longer hospital length of stay, functional and cognitive decline, increased mortality, and notably higher health care costs (4, 5). Delirium is a preventable condition (6), and may be a modifiable risk factor for cognitive decline and dementia in older adults (7, 8).

Previous studies examining the health economic impact of delirium have found that delirium is associated with increased health care costs. Leslie et al. (4), in a cohort of hospitalized patients age 70 + that participated in a clinical trial on delirium prevention and intervention (*n* = 841), found that after adjusting for demographic and clinical characteristics the average costs per day survived among patients with delirium were more than 2.5 times the costs among patients without delirium. Leslie et al. (4) used total inflation-adjusted health care costs (calculated by reimbursed amounts or hospital charges converted to costs from data from Medicare administrative files, hospital billing records, and the Connecticut long-term care registry). Gou et al. (5) examined one-year Medicare costs associated with delirium in older patients undergoing major elective surgery (*n* = 497) and found the cumulative costs attributable to delirium were $44,291 per patient over 1 year; they used the data from the prospective Successful Aging after Elective Surgery (SAGES) study, an ongoing cohort study of older adults undergoing major elective surgery. The study also reported a dose response correlation between increased severity of delirium and higher costs (5). These previous studies were limited in their size, and the study by Gou and colleagues only included participants that underwent surgery.

An emerging area of research, delirium superimposed on dementia, a condition where individuals with pre-existing dementia develop delirium, suggests that healthcare costs are higher when delirium occurs alongside dementia. Hshieh et al. (9), examined one year Medicare costs associated with delirium in hospitalized patients with and without Alzheimer’s Dementia and Related Disorders (ADRD); they analyzed data from the Better Assessment of Illness (BASIL) study, a prospective cohort study of older adults (*n* = 311) hospitalized for a variety of medical issues at a large teaching hospital. They found that in ADRD patients with or without delirium the adjusted mean difference in costs associated with delirium were $34,828 and most of these costs occurred between 90–365 days, while in non-ADRD patients delirium was associated with increased costs at all timepoints (10). Delirium and ADRD are related conditions and there are several studies that have shown that delirium influences cognitive decline in older adults (10, 11). There are limited studies of delirium in hospitalized older adults from unselected populations, and data from nationally representative datasets is sparse (12). Epidemiologic studies can clarify the economic significance of delirium by including more diverse samples and reducing bias. The Medicare Current Beneficiary Survey (MCBS) has been used by other groups to document medical spending in older adults (13, 14).

This study evaluates health care costs attributable to delirium during hospitalization in older Medicare beneficiaries and seeks to increase our understanding of the economic impact of delirium in a nationally representative dataset which expands our understanding of this relationship beyond what has been evaluated previously. This study examines the consequences of delirium beyond hospitalization and its economic impact on patients with dementia and those without dementia. The costs associated with aging are increasing, and delirium, a preventable condition (8), may contribute to this. Additional studies that examine the economic impact of delirium may lead to policy and clinical practice changes with economic benefit.

## 2 Methods

### 2.1 Study population

This study used cross-sectional data from the 2019–2021 Medicare Current Beneficiary Survey (MCBS) research claim files. MCBS is a multipurpose survey conducted in a nationally representative sample of the Medicare population that provides information on demographics, socioeconomic status, expenditures and sources of payment, satisfaction of care, and health outcomes of the study population.

Our analytic sample included a total of *N* = 4,447 participants with at least one hospitalization recorded in the research claims files during the 2019–2021 rounds. Our sample then excluded participants younger than 65 years of age on Medicare due to disability status or severe health conditions like end-stage renal disease (*N* = 726), and those not living in the community (*N* = 329). Further, we excluded all participants with an incomplete set of covariates (*N* = 793 [missing chronic conditions, *N* = 752; stroke diagnosis, *N* = 7; Alzheimer’s and related dementias, *N* = 2; race and ethnicity, *N* = 28; and education, *N* = 4]) yielding an analytic sample of *N* = 2,599 participants corresponding to a total of *N* = 5,677 hospitalizations (Supplementary Figure 1).

MCBS features a representative rotating panel; each panel was interviewed up to three times a year over a four-year period. As a result, when combining the 2019–2021 rounds of MCBS, some participants were observed multiple times across different rounds. MCBS survey and research claim files were obtained from CMS with a data use agreement. As this research used MCBS’ deidentified data, it is exempt from review by the Institutional Review Board and participant consent is not applicable.

### 2.2 Delirium

Delirium was identified in the MCBS data using a claims-based algorithm developed and validated by Kim et al. (15). The algorithm identifies cases of delirium using a series of International Statistical Classification of Disease and Related Health Problems 10th edition (ICD-10 codes) based on all diagnosis codes recorded in the hospitalization claim. As delirium is often underdiagnosed, the algorithm has a high specificity (98%), but low sensitivity (18%) when compared against the criterion standard Confusion Assessment Method (16, 17). For each hospitalization, a binary variable was created for experiencing delirium.

### 2.3 Hospital costs

For each hospitalization claim, we obtained total Medicare payments and total claim charges. Total Medicare payments were computed as the sum of hospital services (henceforth referred to as Medicare hospital payments) plus the number of Medicare-covered days times the per diem payments made by Medicare. Total claim charges correspond to the hospital charges for all services included in the institutional claim. All monetary amounts were deflated to US dollars of 2019 according to the consumer price index for medical care services (18).

### 2.4 Covariates

Covariate selection was guided by *a priori* clinical knowledge of population-level variables associated with delirium and health care costs. Self-reported demographic and socioeconomic variables included age in years, sex (female, male), race ethnicity (White, Black, Hispanic, Asian, and other), education (less than high school, high school graduate, some college or more), and any Medicaid eligibility. Self-report (*Has a doctor ever told you have…?*) health variables included history of stroke, dementia or Alzheimer’s diagnosis, and number of chronic conditions among cancer, COPD/asthma, chronic heart disease, acute myocardial infarction, hypertension, diabetes, arthritis, mental disorder or depression. To account for potential geographical differences, we also included participant’s census region of residence (New England, Middle Atlantic, E North Central, W North Central, South Atlantic, E South Central, W South Central, Mountain, and Pacific). Additionally, the length of stay was computed as the number of days from hospital admission to discharge. For study participants who were observed across multiple rounds, covariates were measured at the first round they appeared in the sample.

### 2.5 Statistical approach

The characteristics of study participants were described by delirium status (if participants ever had hospitalization with delirium), and hospitalizations were described by delirium. Differences by groups were tested using Pearson’s chi-squared tests for categorical variables, and Kruskal-Wallis ANOVA tests for continuous variables. We estimated the excess cost associated with delirium using a mixed-effects linear model with a random intercept at the individual level for Medicare payments (hospital and total payments) and total claim charges separately. Our strategy followed a model building approach in three stages; we first estimated the unadjusted model, we then adjusted for patient demographics (sex, age, race/ethnicity, and education level). Finally, we also included prior diagnosis of stroke, ADRD, number of chronic conditions, length of stay, and fixed effects for census region and survey year.

We also explored the moderating effect of Alzheimer’s disease and related dementias on the association between delirium and hospitalization costs by estimating the fully adjusted model stratified by participant ADRD status and using a model including an interaction term between delirium and ADRD status. Given the importance of different health insurance on determining medical expenditures, additional analyses stratified by Medicaid coverage were also performed.

Given the large number of observations excluded due to missing covariates, we also described participant characteristics by inclusion status in our final analytical sample. In sensitivity analysis, we implemented multiple imputations by chained equations (MICE) approach to deal with the missing covariates before re-estimating our models. All analyses were performed using Stata SE/18.0 statistical software. The threshold for statistical significance was set at *α* = 0.05.

## 3 Results

Our final analytic sample included a total of 2,599 participants (mean age, 78.4 ± 7.7 years, 47.1% female, 81.8% White, 6.6% Black) totaling 5,677 hospitalizations. A total of 286 (11.0%) of participants experienced delirium during a hospitalization, while the rest 2,313 (89.0%) never did. Compared to participants who never experienced delirium, those who experienced at least one delirium episode were older (80.0 vs. 78.2 years), more likely to have less than a high school education (20.3% vs. 14.7), and had a higher number of chronic conditions (2.6 vs. 2.4) (Table 1).

**Table 1:** Characteristics of study participants by delirium.

	Total	No delirium	Delirium	*p*-value
	*N* = 2,599	*N* = 2,313	*N* = 286	*p*-value
Age (y), mean (SD)	78.4 (7.7)	78.2 (7.7)	80.0 (7.9)	<0.001
Education, *N* (%)				0.022
Less than HS	397 (15.3)	339 (14.7)	58 (20.3)	
HS graduate	1,259 (48.4)	1,120 (48.4)	139 (48.6)	
Some college or more	943 (36.3)	854 (36.9)	89 (31.1)	
Sex, *N* (%)				0.860
Female	1,223 (47.1)	1,087 (47.0)	136 (47.6)	
Male	1,376 (52.9)	1,226 (53.0)	150 (52.4)	
Race ethnicity, *N* (%)				0.018
White NH	2,127 (81.8)	1,907 (82.4)	220 (76.9)	
Black NH	172 (6.6)	155 (6.7)	17 (5.9)	
Hispanic	188 (7.2)	161 (7.0)	27 (9.4)	
Asian	42 (1.6)	34 (1.5)	8 (2.8)	
Other	70 (2.7)	56 (2.4)	14 (4.9)	
Any Medicaid coverage, *N* (%)	294 (11.3)	243 (10.5)	51 (17.8)	<0.001
Num. of chronic conditions, mean (SD)	2.4 (1.5)	2.4 (1.5)	2.6 (1.6)	0.030
Num. of hospitalizations, mean (SD)	2.2 (2.0)	2.1 (1.9)	3.1 (2.7)	<0.001
Stroke, *N* (%)	428 (16.5)	349 (15.1)	79 (27.6)	<0.001
Dementia or Alzheimer, *N* (%)	182 (7.0)	121 (5.2)	61 (21.3)	<0.001

MCBS (2019–2021). SD, standard deviation; HS, high school. Number of chronic conditions among cancer, COPD/asthma, chronic heart disease, acute myocardial infarction, hypertension, diabetes, arthritis, mental disorder or depression. Other race includes Native Hawaiian or Pacific Islander, American Indian or Alaska Native, or Multiracial responses. *p*-values correspond to Pearson’s chi-squared (for categorical variables) or Kruskal-Wallis ANOVA tests (for continuous variables). Participants with delirium had to experience at least one hospitalization with delirium. All monetary values presented are in US dollars of 2019.

Among the 5,677 hospital stays analyzed, the average length of stay was 5.2 ± 5.3 days, with average hospital charges totaling $63,679.5. In total, delirium was identified in 506 (8.9%) hospitalizations. Hospitalizations in which delirium was identified were on average longer (8.3 vs. 4.9 days) and were associated with higher costs (total Medicare payment $17,758.3 vs. $12.669.8) than those without delirium (Table 2).

**Table 2:** Characteristics of hospitalizations by delirium.

	Total	No Delirium	Delirium	*p*-value
Mean (SD)	*N* = 5,677	*N* = 5,171	*N* = 506	
Length of stay (d)	5.2 (5.3)	4.9 (4.9)	8.3 (7.5)	<0.001
Total hospital charges (USD)	63,679.5 (77,489.2)	61,417.6 (71,642.9)	86,795.1 (119,810.7)	<0.001
Total Medicare payment (USD)	13,123.3 (13,472.7)	12,669.8 (12,141.2)	17,758.3 (22,525.7)	<0.001
Medicare per diem payment (USD)	416.8 (1,263.6)	380.8 (1,059.6)	785.0 (2,510.4)	<0.001
Medicare hospital payments (USD)	12,706.5 (12,966.2)	12,289.0 (11,784.7)	16,973.3 (21,161.4)	<0.001

MCBS (2019–2021). SD, standard deviation; HS, high school. All monetary values displayed in US dollars of 2019. *P*-values correspond to Pearson’s chi-squared (for categorical variables) or Kruskal-Wallis ANOVA tests (for continuous variables).

In unadjusted models, we found that on average, hospitalizations in which delirium was identified incurred in total hospital charges in excess of $28,828.4 (95% confidence interval, CI: $21,762.7, $35,894.1) when compared to hospitalizations without delirium (Table 3). In fully adjusted models, we found that hospitalizations with delirium incurred in total hospital charges in excess of $8,109.5 (95% CI: $1,859.5, $14,359.5) (Table 3).

**Table 3:** Mixed effect model for association between delirium and excess hospitalization cost.

	Model 1		Model 2		Model 3	
	β (95% CI)	*p*-value	β (95% CI)	*p*-value	β (95% CI)	*p*-value
Total charge amount (USD)						
Delirium						
No delirium	1 ref		1 ref		1 ref	
Delirium	28,828.39 (21,762.74, 35,894.1)	<0.001	29,930.1 (22,896.7, 36,963.5)	<0.001	8,109.5 (1,859.5, 14,359.5)	0.011
Medicare hospital payments (USD)						
Delirium						
No delirium	1 ref		1 ref		1 ref	
Delirium	4,883.8 (3,693.6, 6,074.0)	<0.001	5,056.9 (3,867.7, 6,246.0)	<0.001	1,418.5 (347.0, 2,489.9)	0.009
Total Medicare payments (USD)						
Delirium						
No delirium	1 ref		1 ref		1 ref	
Delirium	5,333.9 (4,097.5, 6,570.4)	<0.001	5,510.4 (4,275,1, 6,745.6)	<0.001	1,630.7 (527.0, 2,734.5)	0.004

MCBS (2019–2021) research claims data files (*N* = 5,677). CI, confidence interval. Excess expenditure estimated using a mixed-effects linear model with a random intercept at the individual level. Model 1 is the unadjusted model; Model 2 includes patient demographics (sex, age, race/ethnicity, and education level) as covariates. Model 3 includes all covariates from model 2 in addition to prior diagnosis of stroke, dementia, number of chronic conditions, length of stay, and survey year. All results presented are in US dollars of 2019.

**Figure 1:**
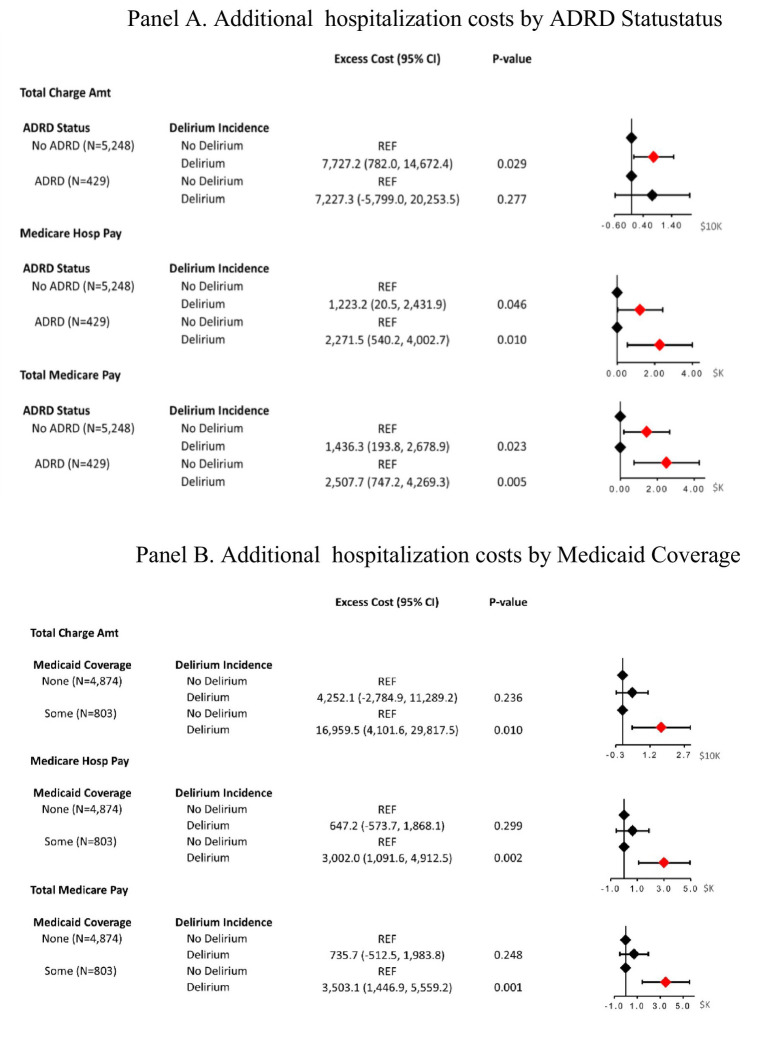
Mixed effect model for association between delirium and excess hospitalization cost. MCBS (2019–2021) research claims data files: subgroup analysis abbreviations: ADRD. Alzheimer’s disease and related dementias; CI, Confidence interval; Amt, amount; Hosp., hospitalization. Excess expenditure estimated in USD using a mixed-effects linear model with a random intercept at the individual level stratified by ADRD status **(A)** and Medicaid coverage **(B)**. All models are adjusted for participant’s sex, age, race/ethnicity, education level, prior diagnosis of stroke, number of chronic conditions, length of stay, and fixed effects for census region and survey year. All results presented are in US dollars of 2019.

With respect to Medicare payments, delirium was associated with higher hospital payments and higher total payments in both the unadjusted and fully adjusted models (Table 3). Specifically, in fully adjusted models, delirium was associated with Medicare hospital payments in excess of $1,418.5 (95% CI: $347.0, $2,489.9) and total Medicare payments in excess of $1,630.8(95% CI: $527.0, $2,734.5). When excess hospitalization costs due to delirium were examined in MCBS participants, we found that delirium is more costly among participants with ADRD than no ADRD history (Figure 1A). These differences did not achieve statistical significance; this may be due to the limited sample size in the ADRD group. In stratified analysis by Medicaid coverage, we found that excess cost was higher among participants with any Medicaid coverage than among those without any Medicaid coverage (Figure 1B).

Compared to participants in our analytic sample, excluded participants (*N* = 793) were on average older (83.8 vs. 78.4 years) and more likely to have some Medicaid coverage (36.4% vs. 11.3%) and more experienced a delirium event (32.9% vs. 11.0) (Supplementary Table S1). Excluded participants had a total of *N* = 1,901 recorded hospitalizations. Hospitalizations from excluded participants were longer (6.3 vs. 5.2 days) and less costly (total hospital charges: $61,058.8 vs. $63,679.5; total Medicare payments: $12,581.9 vs. $13,123.3) (Supplementary Table S2). Sensitivity analyses after imputation for missing variables showed similar, though less robust results (Supplementary Table S3).

## 4 Discussion

In this cohort of hospitalized older Medicare beneficiaries, delirium (identified using claims data), during hospitalization was associated with higher total hospital charges and higher total Medicare payment compared to costs for beneficiaries that did not experience delirium, after adjusting for comorbidities, health conditions, and sociodemographic factors. While prior studies have demonstrated increased costs associated with delirium, most were from studies that included select cohorts of patients with more acute care needs, like the ED and ICU (19–22), which can impact cost calculations. In addition, the vast majority of studies have relied on clinical samples as opposed to broader representative data that include more heterogeneous populations and are helpful to inform policy.

This analysis is one of few to examine the association between delirium and excess hospital costs using data from a nationally representative cohort of hospitalized older adults. In addition, when 1-year health care costs due to delirium were examined by ADRD status, we found that delirium is more costly among participants with ADRD than among those with no ADRD history. Our findings of higher costs associated with delirium are similar to findings from other smaller cohorts (4, 5, 9, 23). Leslie et al. (4), found in their study that patients with delirium costs more than 2.5 times the cost of patients without delirium. Our findings suggest that focusing on delirium prevention and intervention may lead to significant cost savings.

There are notable limitations in this study. The most significant limitation is the ascertainment of delirium episodes from hospital claims data, which likely underreport delirium events (24). Also, the accuracy of delirium ICD coding by clinicians poses a challenge, which may not be as accurate as delirium measures in research studies (25, 26). Moreover, we are also unable to analyze the associated costs with delirium severity or main reason for hospitalization as some hospitalizations are more costly than others (i.e., if associated with surgery/procedure). In addition, our data does not include additional information such as self-reported functional impairments or information on phenotypic frailty, which have been shown to be associated with higher subsequent health care expenditures in community-dwelling beneficiaries (27). Importantly, there was a non-negligible number of missing information on chronic conditions in patients within MCBS with hospitalizations. The patients that were excluded from our main analyses due to missing data were older, more likely to be female and receive Medicaid coverage. However, due to missing information regarding chronic conditions, we cannot necessarily compare health burden across groups, which might explain, the estimates obtained from our MICE analysis, if excluded participants were healthier.

This study has many strengths and there are numerous benefits of using the MCBS. This dataset contains information on hospital length of stay (LOS), which is often longer in individuals with delirium (28). In our sample we found a mean hospital LOS of 8.3 days in patients with delirium vs. 4.5 days in patients without delirium, this can impact associated costs (29), and we were able to account for this difference in our analyses. Also, the MCBS collects information directly from beneficiaries and links their responses to administrative claims; this allowed for additional analyses by ADRD status. The MCBS collects survey-reported data on all sources of payment for health care costs, including those not covered by Medicare, and this allows a complete source of payment information to fully assess costs as described. The MCBS features a rotating panel design that represents the population of all Medicare beneficiaries for the survey year, with each panel interviewed up to three times a year over a four-year period, enabling longitudinal analysis.

In conclusion, delirium during hospitalization was associated with higher one-year health care costs in this nationally representative cohort. Delirium was also associated with higher costs among participants with ADRD than those with no history of ADRD. This work agrees with previous work that delirium is associated with increased hospital costs in older adults. This has significant implications at the hospital level as well as larger policy implications due to larger nation-wide economic impact (30). Moreover, as delirium may be a modifiable risk factor for cognitive decline and dementia in older adults (31), efforts to prevent and manage delirium are greatly needed and may be instrumental in counteracting the increasing costs of caring for older adults. This coincides with recent Centers for Medicare & Medicaid Services (CMS) requirements for delirium screening as part of the Age-Friendly Hospital Measures for the Inpatient Quality Reporting Program (32).

## Data Availability

Publicly available datasets were analyzed in this study. This data can be found at: https://data.cms.gov/Medicare-current-beneficiary-survey-mcbs/Medicare-current-beneficiary-survey-survey-file.
