# Supplementary material for: Additional hospitalization costs associated with delirium among older adults: evidence from the Medicare Current Beneficiary Survey
Source: Front Public Health. 2026 Jan 28;14:1750969. doi: 10.3389/fpubh.2026.1750969 (PMC12891191; doi:10.3389/fpubh.2026.1750969)
Supplement: Supplementary file 1 [file Supplementary_file_1.docx]

# Supplementary Materials

**Supplemental Figure 1**. Flow diagram with participant inclusion and exclusion criteria

Excluded for not community dwelling

N=329

Excluded for age<65

N=726

MCBS 2019-2021

Total participants with atleast 1 hospitalization

N=4,447

Excluded for incomplete covariates

N=793

*Incomplete covariate exclusions:*

*Chronic conditions N=752*

*Stroke diagnosis N=7*

*Alzheimer’s disease and related dementias N=2*

*Race and ethnicity N=28*

*Education N=4*

**Final sample**

**N=2,599**

**Supplemental Figure 2**. Illustration of additional costs due to delirium during hospitalization


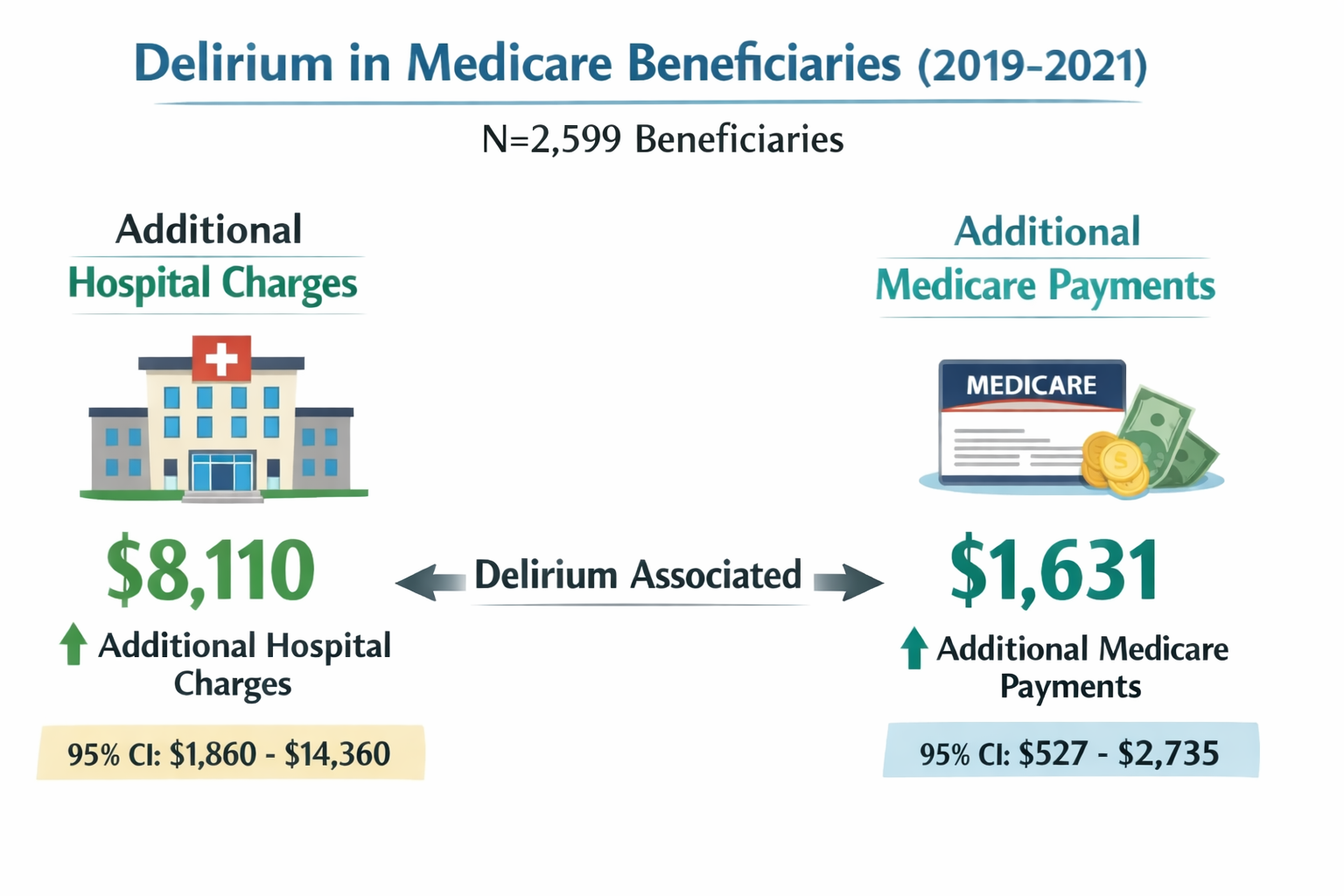


Notes: AI generated image: ChatGPT date 1-6-2026- prompt “generate an image that represents in a sample of 2,599 Medicare beneficiaries (2019- 2021), the occurrence of delirium was associated with $8,110 (95% confidence interval, CI: $1,860, $14,360) additional hospital charges and $1,631(95% CI: $527, $2,735) additional Medicare payments”

**Table S1.** Characteristics of Study Participants by Inclusion in Our Analytic Sample. MCBS (2019-2021)

|  | **Total** | **Excluded** | **Included** | **p-value** |
| --- | --- | --- | --- | --- |
|  | N=3,392 | N=793 | N=2,599 |  |
| **Delirium** |  |  |  | <0.001 |
| Never Delirium | 2,845 (83.9%) | 532 (67.1%) | 2,313 (89.0%) |  |
| Delirium | 547 (16.1%) | 261 (32.9%) | 286 (11.0%) |  |
| **Age** (y), mean (SD) | 79.6 (8.2) | 83.8 (8.5) | 78.4 (7.7) | <0.001 |
| **Education**, N (%) |  |  |  | <0.001 |
| Less than HS | 524 (15.4) | 127 (16.0) | 397 (15.3) |  |
| HS Graduate | 1,544 (45.5) | 285 (35.9) | 1,259 (48.4) |  |
| Some College or More | 1,215 (35.8) | 272 (34.3) | 943 (36.3) |  |
| Missing | 109 (3.2) | 109 (13.7) |  |  |
| **Sex**, N (%) |  |  |  | <0.001 |
| Female | 1,517 (44.7) | 294 (37.1) | 1,223 (47.1) |  |
| Male | 1,875 (55.3) | 499 (62.9) | 1,376 (52.9) |  |
| **Race Ethnicity**, N (%) |  |  |  | <0.001 |
| White | 2,726 (80.4) | 599 (75.5) | 2,127 (81.8) |  |
| Black | 244 (7.2) | 72 (9.1) | 172 (6.6) |  |
| Hispanic | 248 (7.3) | 60 (7.6) | 188 (7.2) |  |
| Asian | 59 (1.7) | 17 (2.1) | 42 (1.6) |  |
| Other | 82 (2.4) | 12 (1.5) | 70 (2.7) |  |
| Missing | 33 (0.9) | 33 (0.9) |  |  |
| **Any Medicaid Coverage**, N (%) | 583 (17.2) | 289 (36.4) | 294 (11.3) | <0.001 |
| **Num. of Hospitalizations**, mean (SD) | 2.4 (1.5) | 2.5 (1.7) | 2.4 (1.5) | 0.660 |

Abbreviations: SD, standard deviation; HS, high school.

Notes: Other race includes Native Hawaiian or Pacific Islander, American Indian or Alaska Native, or Multiracial responses. P-values correspond to Pearson’s chi-squared (for categorical variables) or Kruskal-Wallis ANOVA tests (for continuous variables). All monetary values displayed in US dollars of 2019

**Table S2.** Characteristics of Hospitalizations by Inclusion in Our Analytic Sample. MCBS (2019-2021)

|  | Total | Excluded | Included | p-value |
| --- | --- | --- | --- | --- |
|  | N=7,578 | N=1,901 | N=5,677 |  |
| **Delirium** |  |  |  | <0.001 |
| No Delirium | 6,615 (87.3%) | 1,444 (76.0%) | 5,171 (91.1%) |  |
| Delirium | 963 (12.7%) | 457 (24.0%) | 506 (8.9%) |  |
| **Length of Stay (d)** | 5.5 (5.6) | 6.3 (6.4) | 5.2 (5.3) | <0.001 |
| **Total Hospital Charges** (USD) | 63,022.1 (90,055.6) | 61,058.8 (119,995.1) | 63,679.5 (77,489.2) | 0.27 |
| **Total Medicare Payment** (USD) | 12,987.5 (13,434.2) | 12,581.9 (13,313.8) | 13,123.3 (13,472.7) | 0.13 |
| **Medicare per Diem Payment** (USD) | 420.1 (1,231.2) | 429.9 (1,129.3) | 416.8 (1,263.6) | 0.69 |
| **Medicare Hospital Payments** (USD) | 12,567.4 (12,971.4) | 12,152.0 (12,981.6) | 12,706.5 (12,966.2) | 0.11 |

Abbreviations: SD, standard deviation; HS, high school.

Notes: All monetary values displayed in US dollars of 2019. P-values correspond to Pearson’s chi-squared (for categorical variables) or Kruskal-Wallis ANOVA tests (for continuous variables).

**Table S3.** Sensitivity Analysis Using Multiple Imputations by Chain Equations. Mixed Effect Model for Association between Delirium and Excess Hospitalization Cost. MCBS (2019-2021) Research Claims Data Files (N=7,617)

|  | **Model 1** | | | **Model 2** | | | **Model 3** | |
| --- | --- | --- | --- | --- | --- | --- | --- | --- |
|  | **β (95% CI)** | **p-value** | | **β (95% CI)** | **p-value** | | **β (95% CI)** | **p-value** |
|  |  | | | | | | | |
|  | Total Charge Amount | | | | | | | |
| **Delirium** |  | |  |  | |  |  |  |
| No Delirium | 1 REF | |  | 1 REF | |  | 1 REF |  |
| Delirium | 24,365.2 (18,230.9, 30,499.5) | | <0.001 | 26,511.0 (20,378.9, 34,643.2) | | <0.001 | 4,474.0 (-985.5, 9,933.4) | 0.108 |
|  |  | |  |  | |  |  |  |
|  | Medicare Hospital Payments | | | | | | | |
| **Delirium** |  | |  |  | |  |  |  |
| No Delirium | 1 REF | |  | 1 REF | |  | 1 REF |  |
| Delirium | 3,973.6 (3,091.0, 4,856.2) | | <0.001 | 4,230.3 (3,344.6, 5,116.1) | | <0.001 | 826.7 (51.7, 1,601.8) | 0.037 |
|  |  | |  |  | |  |  |  |
|  | Total Medicare Payments | | | | | | | |
| **Delirium** |  | |  |  | |  |  |  |
| No Delirium | 1 REF | |  | 1 REF | |  | 1 REF |  |
| Delirium | 4,350.7 (3,437.0, 5,264.4) | | <0.001 | 4,604.3 (3,687.5, 5,521.1) | | <0.001 | 1,021.2 (225.0, 1,817.5) | 0.012 |

Abbreviations: CI, confidence interval.

Notes: Excess expenditure estimated using a mixed-effects linear model with a random intercept at the individual level. This sensitivity analysis was completed after Multiple Imputation by Chain Equations. Model 1 is the unadjusted model; Model 2 includes patient demographics (sex, age, race/ethnicity, and education level) as covariates. Model 3 includes all covariates from model 2 in addition to prior diagnosis of stroke, ADRD, number of chronic conditions, length of stay, and fixed effects for census region and survey year. All results presented in US dollars of 2019.
